# Supplementary material for: Reconstitution of prospermatogonial specification in vitro from human induced pluripotent stem cells
Source: Nat Commun. 2020 Nov 9;11:5656. doi: 10.1038/s41467-020-19350-3 (PMC7653920; doi:10.1038/s41467-020-19350-3)
Supplement: Supplementary file 1 — Supplementary Information [file 41467_2020_19350_MOESM1_ESM.pdf]

Inventory of Supplementary Information

SUPPLEMENTARY FIGURES (Supplementary Fig. 1-8)

Supplementary Fig. 1. Identification of cell subsets and their markers in human fetal testes

Supplementary Fig. 2. Generation of AGVTPC-knock-in hiPSCs

Supplementary Fig. 3. Emergence of GCs with features characteristic of M-prospermatogonia in d77 xrTestes

Supplementary Fig. 4. Characterization of GCs derived from hiPSCs

Supplementary Fig. 5. Dissociation-induced gene expression in *in vitro*-derived cells

Supplementary Fig. 6. DEGs by pairwise comparison between *in vitro*-derived cells

Supplementary Fig. 7. Key markers of *in vitro*-derived GCs compared with *in vivo* GCs

Supplementary Fig. 8. Comparison of transcriptomes between cell clusters in this study and neonatal prospermatogonia

SUPPLEMENTARY DATASETS, see separate Excel documents

Supplementary Dataset 1. DEGs between cell clusters in fetal testes

Supplementary Dataset 2. DEGs between cell clusters *in vitro*

Supplementary Dataset 3. DEGs between T1 and T1LCs

Supplementary Dataset 4. Markers for migrating, mitotic and mitotic-arrest male FGCs defined by Li et.al. 2017

Supplementary Dataset 5. Comparison of gene expression between T1LCs in this study and ag120AG$^{+/-}$VT^{+} cells by Yamashiro et al. 2018

Supplementary Dataset 6. Primers used in this study

Supplementary Dataset 7. Antibodies used in this study

Supplementary Figure 1

a

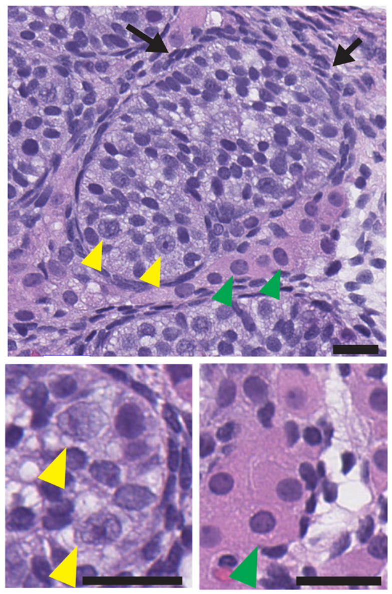

b

	Total Reads	Mean Reads/cell	Median gene/cell	Detected gene	Captured cell	Sequencing Saturation
Hs26-18W0d	177,303,671	40,508	2,710	27,720	4,377	36.4%
Hs27-18W5d	136,780,127	22,586	1,888	26,194	6,056	27.0%
HS31-17W3d	415,576,558	69,309	2,740	26,845	5,996	60.6%

d

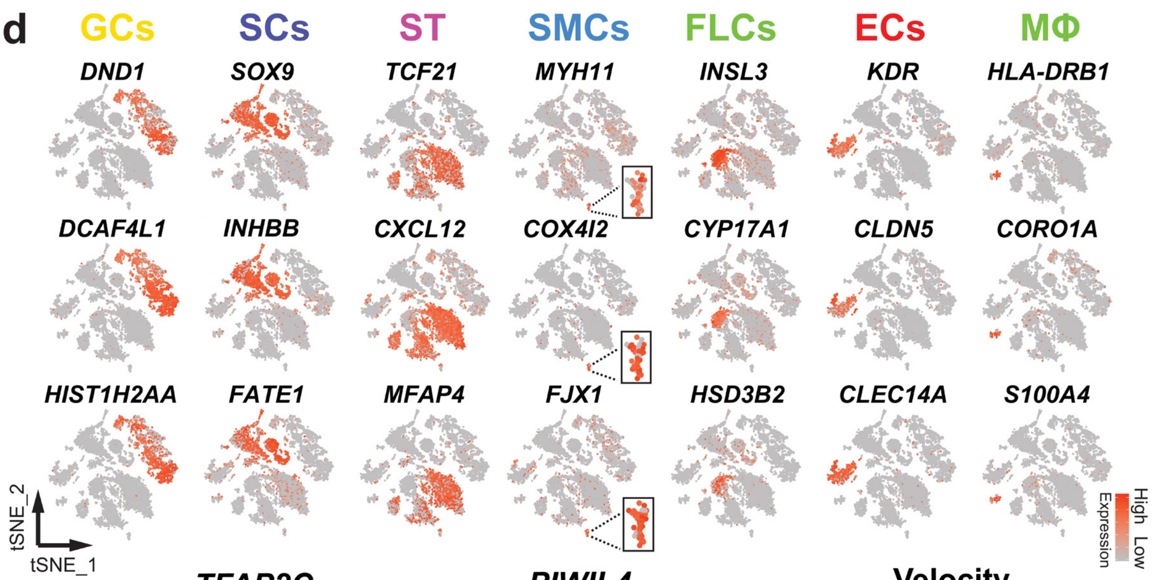

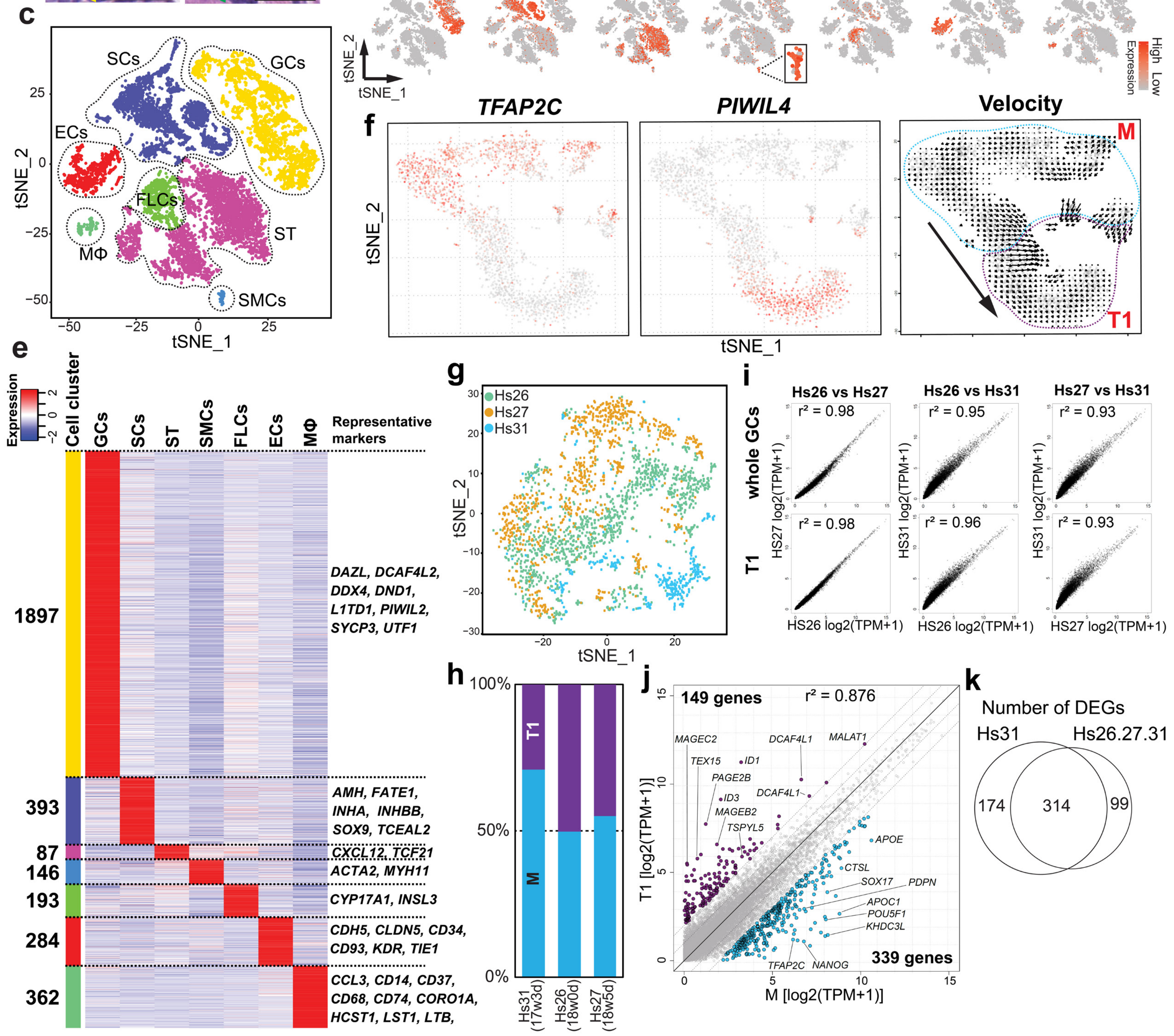

Supplementary Fig. 1. Identification of cell subsets and their markers in human fetal testes

a A fetal testis at 17w3d (Hs31) (top, low magnification) and 18w0d (Hs26) (bottom, high magnification) stained with hematoxylin and eosin. Yellow arrowheads indicate presumptive GCs. Green arrowheads indicate fetal Leydig cells. The stroma also contains spindle cells of uncertain origin (black arrow). Scale bar, 25 μm.

b Mapping results of *in vivo* samples analyzed by scRNA-seq.

c tSNE plot of all testicular cells in our dataset. Cell cluster identities are determined by feature plots of known markers shown in (**d**).

d Gene expression pattern of marker genes for the indicated cell clusters projected on tSNE plots in (**c**).

e Heatmap of markers for the indicated cell clusters. Cell clusters identified in (**c**) were used for DEG analysis to identify markers for each cell cluster. Representative markers are shown on the right. The number of DEGs for each cell cluster are shown on the left.

f RNA velocity analysis of GCs defined in (**c**). Cluster identifies on this tSNE plot were determined by projecting the expression patterns of *TFAP2C* and *PIWIL4* on the tSNE space (left). The lineage trajectory predicted by RNA velocity analysis is shown as arrows over each plot. Dotted lines circumscribe cells representing M (blue) or T1 (purple). A large arrow indicates the overall direction of the individual arrows at the border between M and T1.

g tSNE plot from Fig. 1a with cells colored according to their sample origin.

h Bar graph showing the relative proportion of M and T1 in each sample.

i Scatter plot comparisons of transcriptome data between each testicular sample (Hs26, Hs27, and Hs31). Transcriptome comparisons are shown for whole GCs (top) and for T1 (bottom).

j Scatter plot comparison of the averaged expression values between M and T1 for Hs31 only. Purple, genes upregulated in T1 (149 genes); cyan, genes upregulated in M (339 genes). Key DEGs are annotated.

k Venn diagram showing the overlap of DEGs (M versus T1 comparison) for Hs31 only and for all three *in vivo* samples.

Supplementary Figure 2

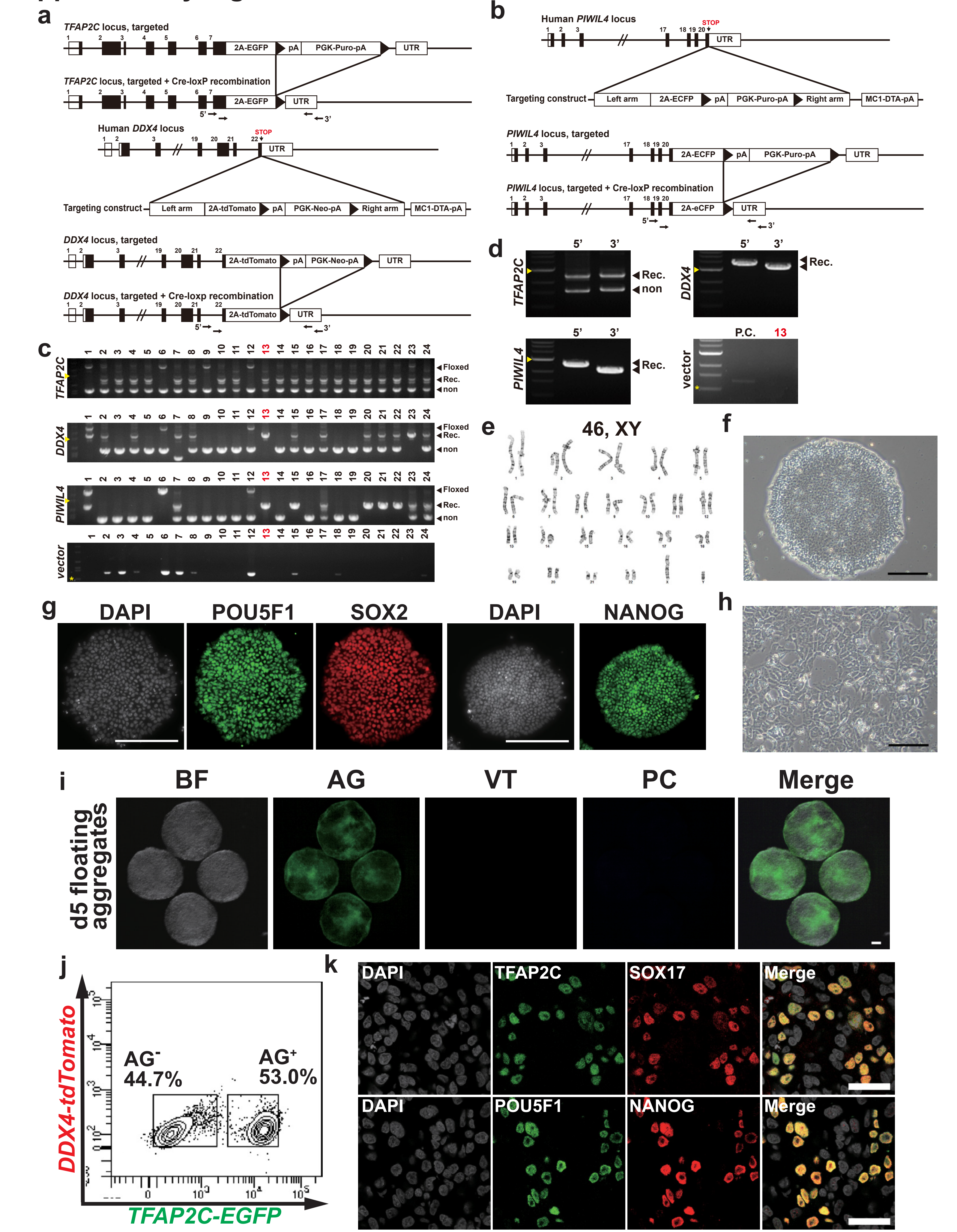

Supplementary Fig. 2. Generation of AGVTPC-knock-in hiPSCs

a Targeting schemes for human *TFAP2C* (top) and *DDX4* (bottom) loci. The *TFAP2C* locus in AG hiPSCs (585B1 1-7) has already been targeted by the *2A-EGFP* knock-in construct (Sasaki et al., 2015[14]) and was recombined by Cre over-expression in this study to remove the Puromycin selection cassette. The construct for knocking-in *2A-tdTomato* into the *DDX4* loci is also shown. Black boxes indicate the exons. Arrows indicate PCR screening primers.

b Schematic illustration of the human *PIWIL4* locus and the construct for knocking-in *2A-ECFP* into the locus.

c PCR screening of the homologous recombinants for *TFAP2C-2A-EGFP* (AG) (top), *DDX4-2A-tdTomato* (VT) (second), *PIWIL4-2A-ECFP* (PC) (third), and of random integration of the targeting vector (bottom). Rec., recombined by Cre; non, non-targeted; Yellow arrow, 3kb; yellow asterisk, 1kb.

d PCR genotyping of the genomic DNA isolated from 9A13 hiPSCs for *AG*, *VT* or *PC* alleles and for random integration of the targeting vector. Yellow arrow, 3kb; yellow asterisk, 1kb. P.C., positive control. Clone 13 (red) was selected for the AGVTPC hiPSC line (9A13).

e Representative result for the 9A13 hiPSC karyotype analysis, showing a normal karyotype (46, XY).

f A phase-contrast image of 9A13 hiPSCs. Bar, 200 μm.

g Immunofluorescence (IF) analysis of 9A13 hiPSCs for DAPI (white), POU5F1 (green, left), SOX2 (red) and NANOG (green, right). Data are representative of two independent experiments. Bars, 20 μm.

h A phase-contrast image of 9A13 hiPSC-derived iMeLCs. Bar, 200 μm.

i Bright-field and fluorescence images of hPGCLCs derived from 9A13 hiPSCs for AG, VT, and PC, with a merge. Bar, 200 μm.

j Fluorescence-activated cell sorting (FACS) analysis of d5 hPGCLCs derived from 9A13 hiPSCs. The percentages of cells in the indicated gates are shown.

k IF images of 9A13 hiPSC-derived d5 hPGCLCs (frozen sections) for TFAP2C (green), SOX17 (red), POU5F1 (green), NANOG (red) and DAPI (white). Merges are shown on the right. Data are representative of two independent experiments. Bars, 20 μm.

Source data are provided as a Source Data file.

Supplementary Figure 3

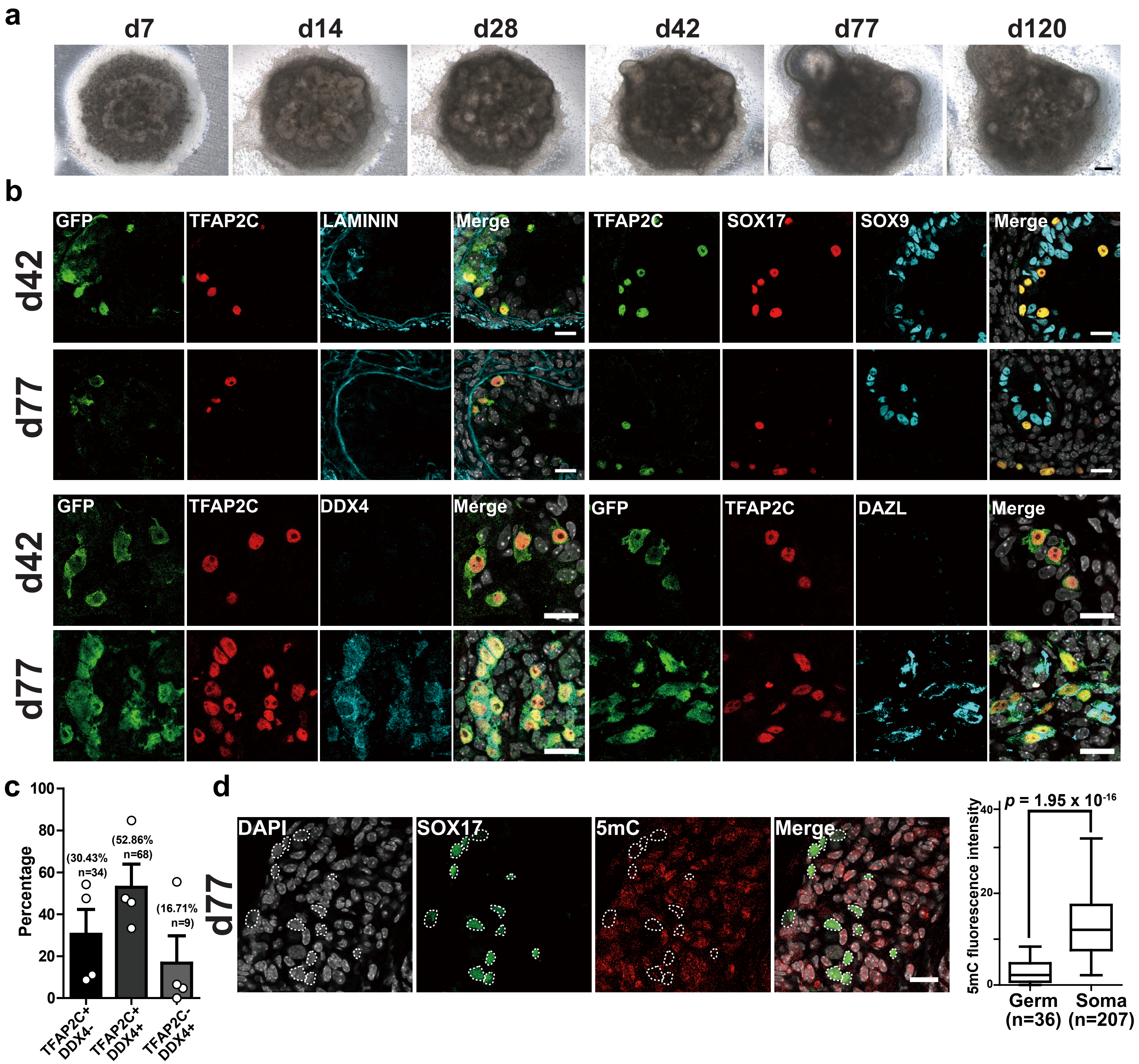

Supplementary Fig. 3. Emergence of GCs with features characteristic of M-prospermatogonia in d77 xrTestes

a Bright-field images of xrTestes from day (d) 7 to d120 in the air-liquid interphase (ALI) culture. Bar, 200 μm.

b Immunofluorescence (IF) analysis of indicated key proteins (green: GFP and TFAP2C, red: TFAP2C and SOX17) with LAMININ or SOX9 (cyan) (top) and of GC markers (red: TFAP2C, cyan: DDX4 and DAZL) in hPGCLC-derived cells (green: GFP) (bottom) in d42 and d77 xrTestes. Bars, 20 μm.

c The proportion of $TFAP2C^{+}DDX4^{-}$, $TFAP2C^{+}DDX4^{+}$, or $TFAP2C^{-}DDX4^{+}$ cells in d77 xrTestes as assessed by IF. Percentages with SEM are per section. Each dot represents the proportion in one section. n, the total number of respective cells in all four sections.

d IF analysis (left) and a box plot showing the fluorescence intensity (right) of 5-methylcytosine (5mC, red) in hPGCLC-derived cells (green: SOX17) and other somatic cells counterstained with DAPI (white). Bar, 20 μm. hPGCLC-derived cells are outlined by white dotted lines. Center line, median; box limits, upper and lower quartiles; whiskers, 1.5× interquartile range. Statistically significant differences between GCs (Germ) and somatic cells (Soma) were identified by two-sided *t*-test assuming unequal variances. $P = 1.95 \times 10^{-16}$. n, the number of respective cells counted.

Source data are provided as a Source Data file.

Supplementary Figure 4

a

	Total Reads	Mean Reads/cell	Median gene/cell	Detected gene	Captured cell	Sequencing Saturation
hiPSCs	85,190,009	68,207	5,973	21,006	1,249	24.3%
iMeLCs	92,135,422	107,761	6,843	21,255	855	23.2%
hPGCLCs_1	107,842,993	87,677	5,432	21,736	1,230	31.0%
hPGCLCs_2	34,961,514	84,245	3,190	18,020	415	48.3%
d81_xrTestis	87,498,123	69,943	5,010	23,123	1,251	32.6%
d124_xrTestis	93,762,487	272,565	6,980	24,737	344	63.6%

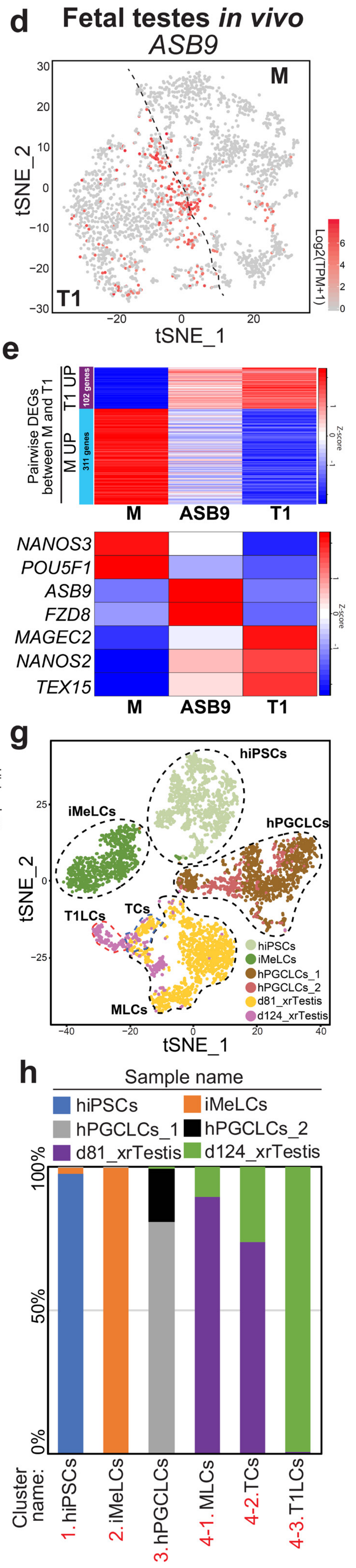

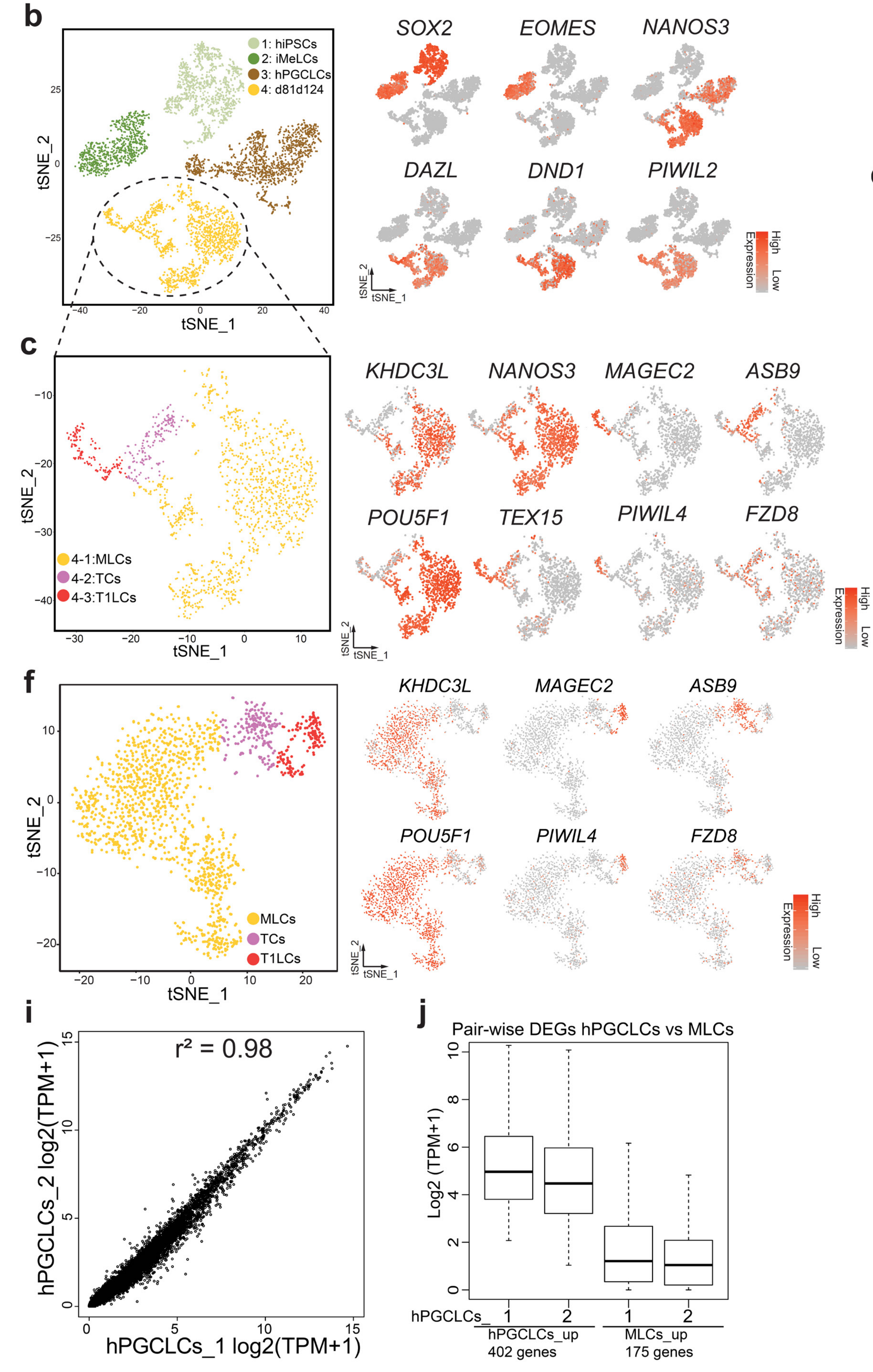

Supplementary Fig. 4. Characterization of GCs derived from hiPSCs
a Mapping results of six *in vitro* samples analyzed by scRNA-seq in this study.
b tSNE plot of *in vitro* derived GCs (same as Fig. 4a) colored based on the four main clusters (left). Cluster identities were determined based on sample origin and by projecting the expression of known markers on the tSNE plot (right). The dotted line denotes the cluster (cluster 4) used for subclassification in (**c**).
c tSNE plot for the subclassification of cluster 4 from (**b**). Three sub-clusters (4-1, 4-2, 4-3) were newly annotated within this cluster and colored accordingly (left). Cluster identities were determined by expression levels of markers for M (*KHDC3L*, *POU5F1*, *NANOS3*) and T1 (*TEX15*, *MAGEC2*, *PIWIL4*) defined in Fig. 1a. Newly identified markers (*ASB9*, *FZD8*) for transitional cells (TCs; cluster 4-2) are also shown (right).
d Expression of *ASB9*, a marker for TCs, projected onto the tSNE plot of *in vivo* testicular samples used in Fig. 1a. The dotted line denotes the border between the M and T1 cell clusters.
e Heatmap of the averaged expression values of genes that were differentially expressed between M and T1 (defined in Fig.1f) (top) or key markers (bottom) in indicated cell types. M and T1 were redefined as the respective cell types defined in Fig.1a, excluding cells expressing *ASB9*. ASB9 denotes cells expressing *ASB9* among the cells in Fig.1a.
f Re-clustering of cluster 4, defined in (**b**), projected on the tSNE plot used for RNA velocity analysis in Fig. 4b (left). Expression levels of markers for M, T1 and TCs are projected on the tSNE plot (right).
g tSNE plot of *in vitro*-derived GCs colored based on sample origin. Dotted lines circumscribe cell clusters defined in (**b**) and (**c**).
h Bar graph showing the proportion of samples in each cell cluster defined in (**b**) and (**c**).
i Scatter plot comparison of averaged transcriptome values for two biological replicates of hPGCLCs (hPGCLCs_1, hPGCLCs_2).
j Box plot of the averaged expression values of genes that were differentially expressed between hPGCLCs and MLCs in two biological replicates of hPGCLC samples.

Supplementary Figure 5

a

	Dissociation-induced genes (van den Brink et al., 2017)	
**Rank**	**Mouse gene symbol**	**Human gene symbol**
1	*Fos*	*FOS*
2	*Hspa1a*	*HSPA1A*
3	*Jun*	*JUN*
4	*Fosb*	*FOSB*
5	*Junb*	*JUNB*
6	*Egr1*	*EGR1*
7	*Hspa1b*	*HSPA1B*
8	*Ubc*	*UBC*
9	*Zfp36*	*ZFP36*
10	*Hspb1*	*HSPB1*
11	*Hsp90aa1*	*HSP90AA1*
12	*Mt2*	*MT2A*
13	*Dnajb1*	*DNAJB1*
14	*Btg2*	*BTG2*
15	*Nr4a1*	*NR4A1*
16	*Cebpd*	*CEBPD*
17	*Hspa8*	*HSPA8*
18	*Mt1*	*MT1A*
19	*Ier2*	*IER2*
20	*Dnaja1*	*DNAJA1*
21	*Socs3*	*SOCS3*
22	*Atf3*	*ATF3*
23	*Jund*	*JUND*
24	*Cebpb*	*CEBPB*
25	*Id3*	*ID3*
26	*Ppp1r15a*	*PPP1R15A*
27	*Hspe1*	*HSPE1*
28	*Cxcl1*	*CXCL1*
29	*Dusp1*	*DUSP1*
30	*Hsp90ab1*	*HSP90AB1*

b

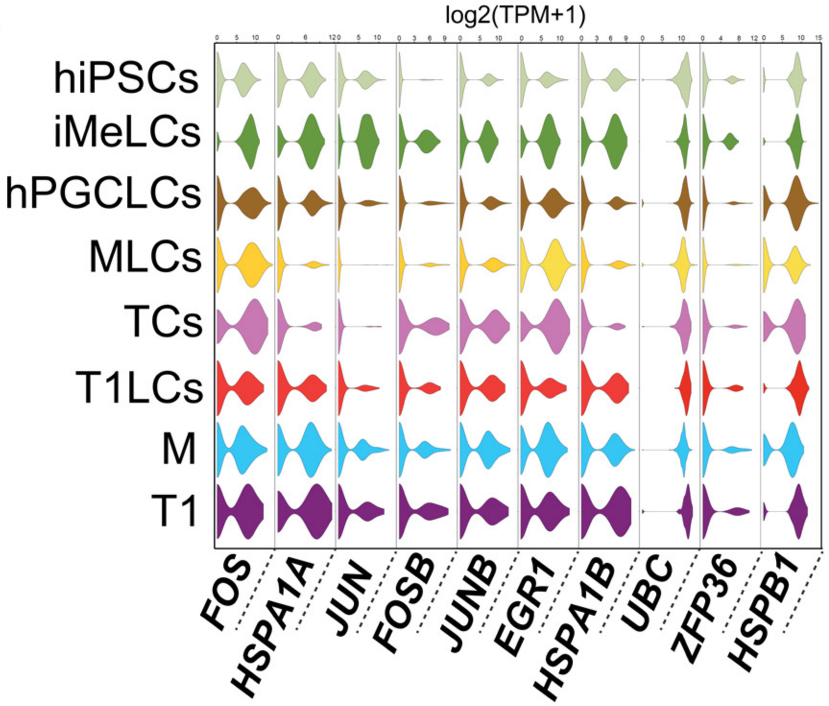

c

Dissociation-induced genes found in DEG lists (ranking in DEG lists sorted by FDR value in ascending order)							
In vitro samples						*In vivo* samples	
hiPSCs	iMeLCs	hPGCLCs	MLCs	TCs	T1LCs	M	T1
NA	*JUN* (127/331)	*BTG2* (200/239)	*NA*	*EGR1* (7/11)	*NR4A1* (739/1268)	*NA*	*HSPB1* (92/102)
	CEBPB (200/331)	*PPP1R15* (208/239)			*DNAJB1* (1211/1268)		*ID3* (10/102)
	HSPA1A (240/331)	*SOCS3* (239/239)					
	HSPA1B (303/331)						

Supplementary Fig. 5. Dissociation-induced gene expression in *in vitro*-derived cells
a Top 30 dissociation-induced genes defined in van den Brink et al., 2017[33].
b Violin plot showing the expression of the top ten dissociation-induced genes in *in vitro* cell clusters.
c Dissociation-induced genes included among respective DEGs. DEGs were defined using a multi-group comparison for *in vitro* clusters (defined in Fig. 4e) and a pairwise comparison between M and T1 (defined in Fig. 1f). The rank of each gene, defined as the position of each gene in the DEG list sorted by ascending FDR value, is shown in parentheses.

Supplementary Figure 6

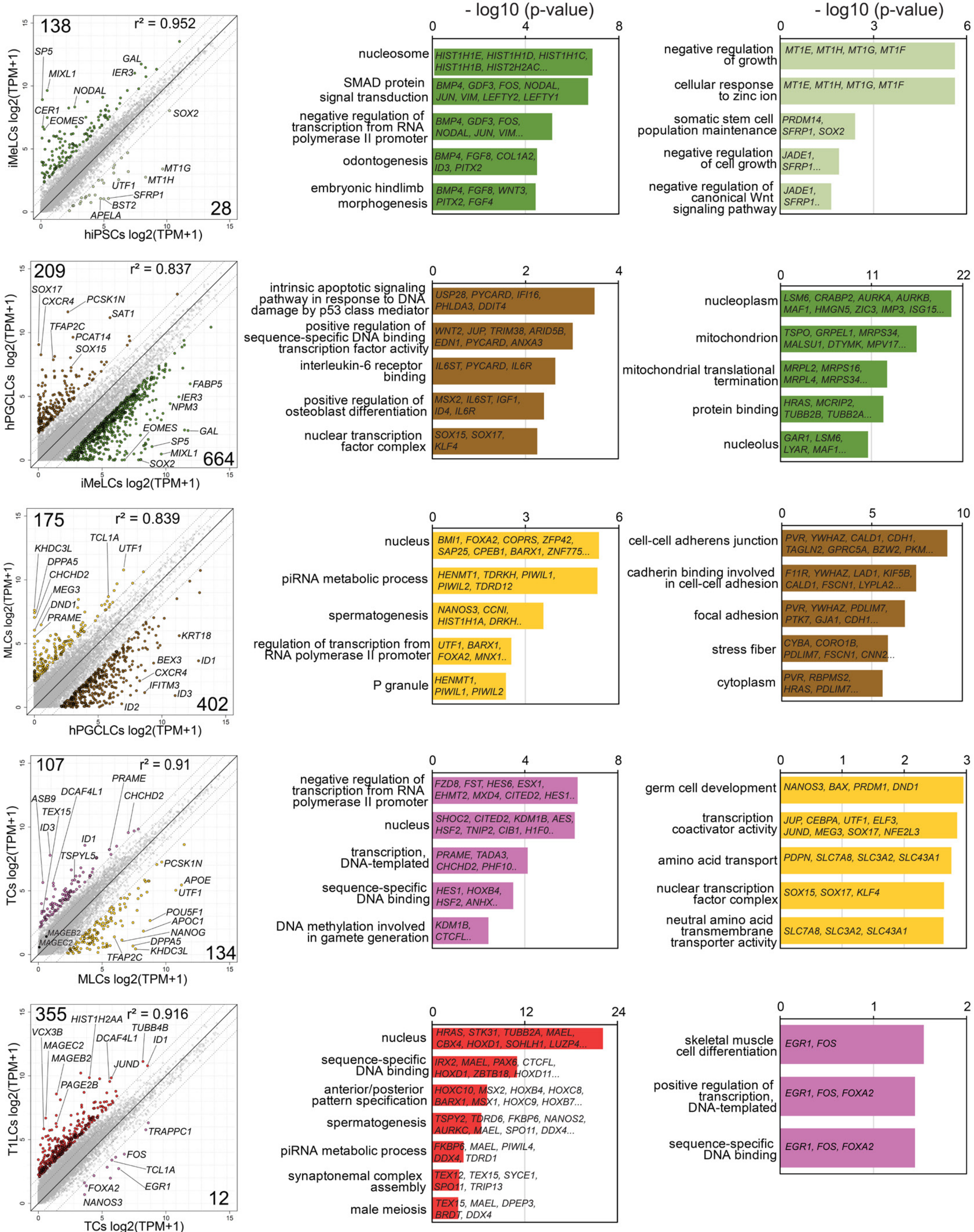

Supplementary Fig. 6. DEGs by pairwise comparison between *in vitro* derived cells
(left) Scatter plots showing DEGs between indicated samples. Genes upregulated by more than 4-fold (FDR < 0.01) in the indicated cell clusters are plotted using the colors in Fig. 4a. Key genes, the number of DEGs, and the coefficient of determination (r^2) are shown. (right) GO analyses of the DEGs. Representative genes in each GO category are shown.

Suppementary Figure 7

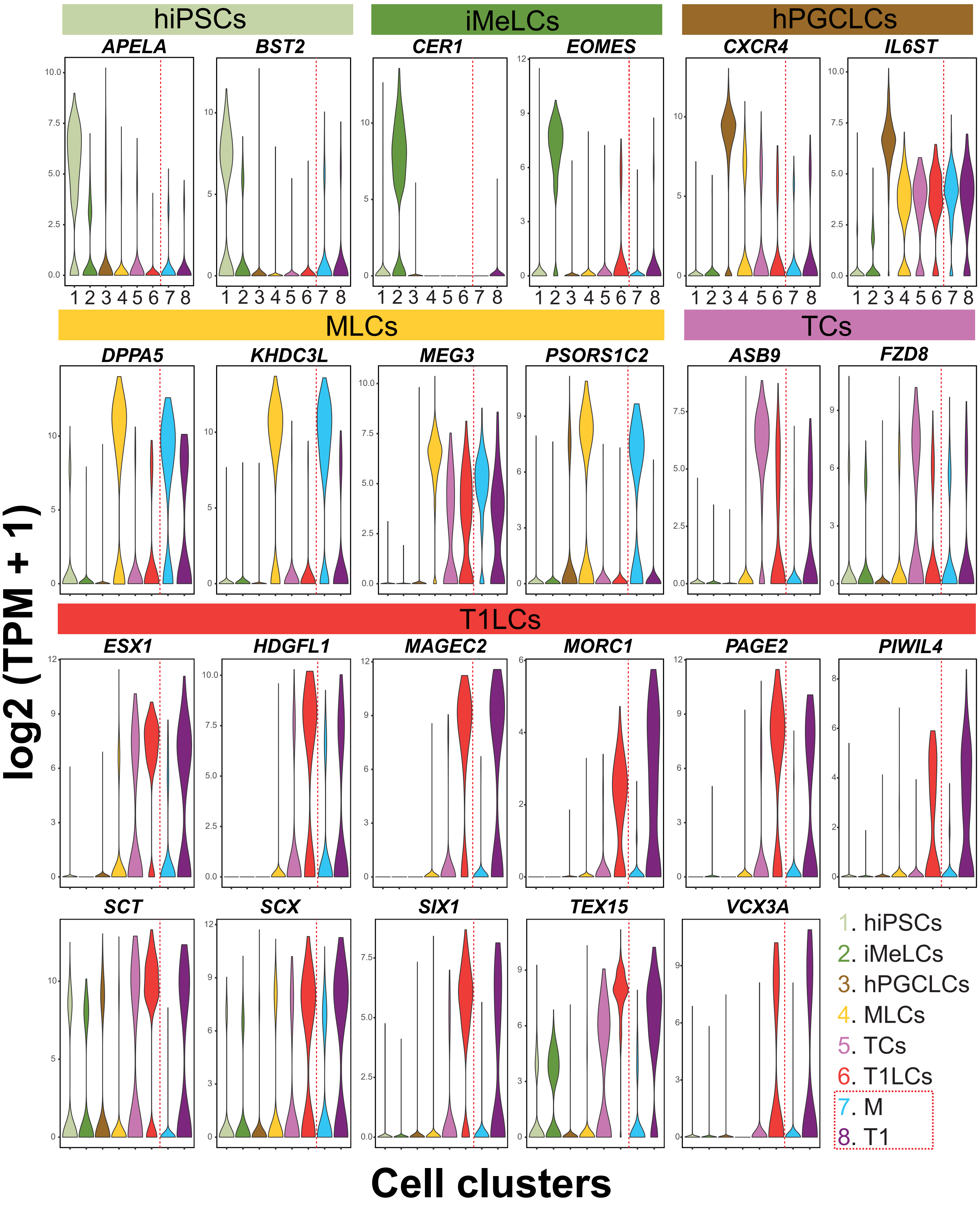

Supplementary Fig. 7. Key markers of *in vitro*-derived GCs compared with in vivo GCs
Violin plots showing the expression levels of key DEGs, defined in Fig. 4e, in the indicated cell clusters. Expression levels of the same genes in in vivo testicular GC clusters (M and T1) are shown on the right for comparison. Color codes for cell clusters are same as Fig. 1a and 4a.

Supplementary Figure 8

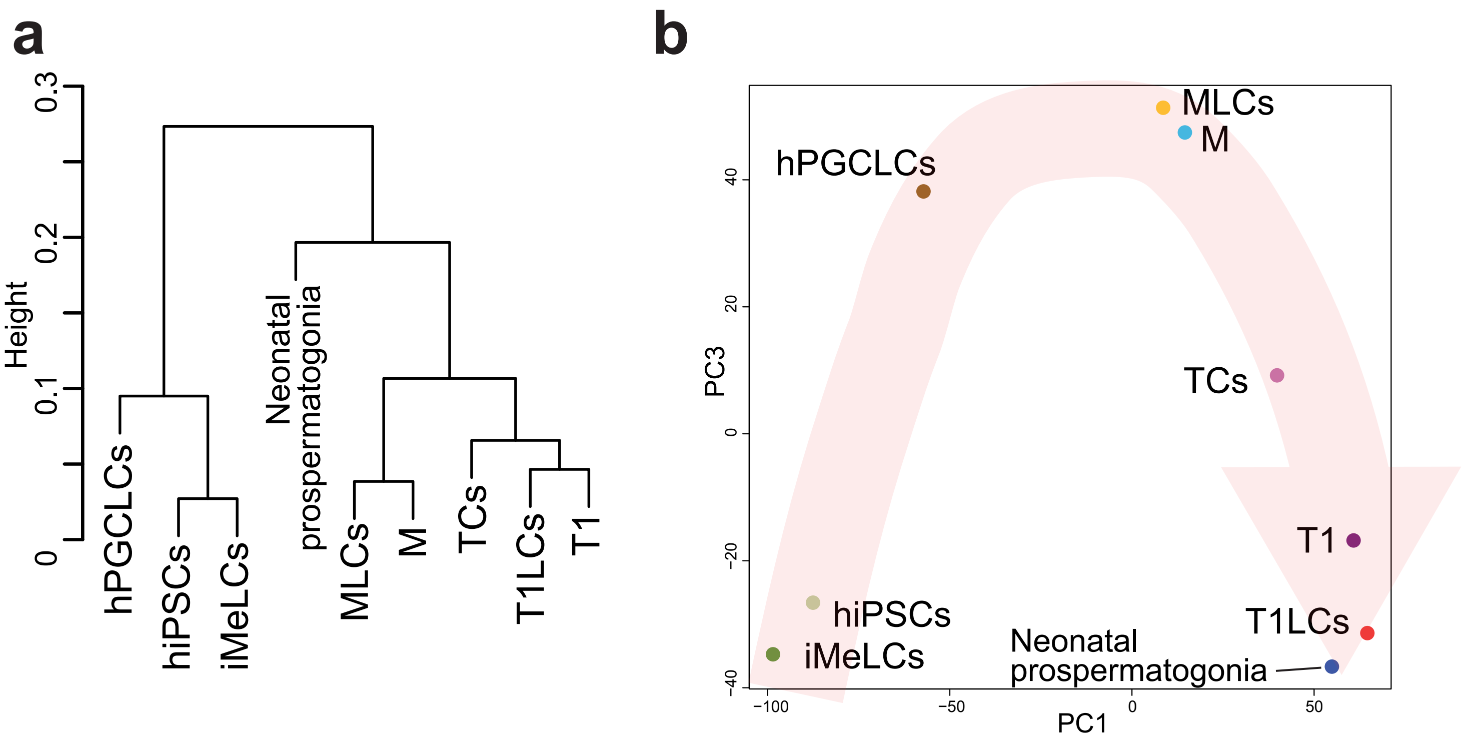

Supplementary Fig. 8. Comparison of transcriptomes between cell clusters in this study and neonatal prospermatogonia

a UHC of the averaged transcriptome values for cell clusters in this study and for neonatal prospermatogonia used in Sohni et al., 2019[18].

b PCA analysis of cells as in (a). Color codes for cell clusters are indicated.
