# Supplementary material for: Reconstitution of prospermatogonial specification in vitro from human induced pluripotent stem cells
Source: Nat Commun. 2020 Nov 9;11:5656. doi: 10.1038/s41467-020-19350-3 (PMC7653920; doi:10.1038/s41467-020-19350-3)
Supplement: Supplementary file 3 — Description of Additional Supplementary Files [file 41467_2020_19350_MOESM3_ESM.pdf]

Description of Additional Supplementary Files

Title: Supplementary Dataset 1.

Description: DEGs between cell clusters in fetal testes

Title: Supplementary Dataset 2.

Description: DEGs between cell clusters in vitro

Title: Supplementary Dataset 3.

Description: DEGs between T1 and T1LCs

Title: Supplementary Dataset 4.

Description: Markers for migrating, mitotic and mitotic-arrest male FGCs defined by Li et.al. 2017

Title: Supplementary Dataset 5.

Description: Comparison of gene expression between T1LCs in this study and ag120AG+/-VT+ cells by Yamashiro et al. 2018

Title: Supplementary Dataset 6.

Description: Primers used in this study

Title: Supplementary Dataset 7.

Description: Antibodies used in this study
