# Supplementary material for: Reconstitution of prospermatogonial specification in vitro from human induced pluripotent stem cells
Source: Nat Commun. 2020 Nov 9;11:5656. doi: 10.1038/s41467-020-19350-3 (PMC7653920; doi:10.1038/s41467-020-19350-3)
Supplement: Supplementary file 11 — Reporting Summary [file 41467_2020_19350_MOESM11_ESM.pdf]

nature research

Corresponding author(s): Kotaro Sasaki

Last updated by author(s): Sep 29, 2020

Reporting Summary

Nature Research wishes to improve the reproducibility of the work that we publish. This form provides structure for consistency and transparency in reporting. For further information on Nature Research policies, see our Editorial Policies and the Editorial Policy Checklist.

Statistics

For all statistical analyses, confirm that the following items are present in the figure legend, table legend, main text, or Methods section.

n/a	Confirmed	
☐	☒	The exact sample size (*n*) for each experimental group/condition, given as a discrete number and unit of measurement
☐	☒	A statement on whether measurements were taken from distinct samples or whether the same sample was measured repeatedly
☐	☒	The statistical test(s) used AND whether they are one- or two-sided<br>*Only common tests should be described solely by name; describe more complex techniques in the Methods section.*
☒	☐	A description of all covariates tested
☐	☒	A description of any assumptions or corrections, such as tests of normality and adjustment for multiple comparisons
☐	☒	A full description of the statistical parameters including central tendency (e.g. means) or other basic estimates (e.g. regression coefficient) AND variation (e.g. standard deviation) or associated estimates of uncertainty (e.g. confidence intervals)
☐	☒	For null hypothesis testing, the test statistic (e.g. *F*, *t*, *r*) with confidence intervals, effect sizes, degrees of freedom and *P* value noted<br>*Give P values as exact values whenever suitable.*
☒	☐	For Bayesian analysis, information on the choice of priors and Markov chain Monte Carlo settings
☐	☒	For hierarchical and complex designs, identification of the appropriate level for tests and full reporting of outcomes
☒	☐	Estimates of effect sizes (e.g. Cohen's *d*, Pearson's *r*), indicating how they were calculated

Our web collection on statistics for biologists contains articles on many of the points above.

Software and code

Policy information about availability of computer code

Data collection All FACS data were collected using FACSDiva software (v8.0.2). Confocal images were captured using Leica LasX (v.3.7.2).

Data analysis Confocal images were quantified in ImageJ. Raw data were demultiplexed using mkfastq command in cell Ranger (v3.1.0) to generate Fastq files. Fastq files were trimmed using cutadapt (v.2.6). Trimmed sequence files were mapped on the reference genome for humans (GRCh38-3.0.0). Read counts were obtained from outputs made by CellRanger (v3.1.0) using edgeR (v3.22.3). Cells with fewer than 1800 genes, and genes with fewer than 10 reads, were filtered out. Genes detected in only five or fewer cells were also omitted from the downstream analyses. Secondary data analyses were performed using R software version (3.6.1) with the ggplot2 (v3.2.1), gplots (ver.3.0.1.1), qvalue (ver.2.18.0), and sp (ver.1.4-1), TCC (v1.12.1) packages and Excel (Microsoft). All analyses of expression data were performed using log2(TPM+1) values. UHC was performed using hclust function with Euclidian distances and Ward's method (ward.D2) or complete method (complete). PCA was performed using the prcomp function without scaling. tSNE analysis was performed using Rtsne function and visualized using ggplot2. The RNA velocity analysis was performed using Velocyte (v.0.6) (http://velocyto.org/) on the cells filtered using the same selection criteria described above.

To identify genes that were differentially expressed among cell types (i.e., marker genes), we used the edgeR function from the TCC package to isolate genes with an FDR < 0.05. The DEGs were then defined as genes exhibiting > 2-fold higher expression in one cluster compared to the remaining clusters. DEGs for each pairwise comparison among cell types were defined as genes with a minimum mean abundance of log2 (TPM+1) > 2, fold-change ≥ 4, and an FDR < 0.01 following Welch's t-test with the Benjamini-Hochberg correction. These DEGs were plotted over the scatter plot of averaged transcriptome values for cell clusters. DEGs were mapped to GO terms using DAVID (v.6.8) with the background list set to "Homo sapiens." Only enriched GO terms with p < 0.05 were shown.

To quantify the expression level of TEs at the locus resolution, we first prepared a transcript annotation file (i.e., GTF file) including human genes and TEs, annotated by RepeatMasker (http://www.repeatmasker.org), as previously described. Read mapping and counting were performed using Cell Ranger with the above annotation file. TE loci detected in ≥ 0.5% of the cells were used in the downstream analyses. The read abundances of total TEs, respective TE families, and TE subfamilies were calculated by summing up the read counts of TE loci. The expression level of TEs was normalized as log2-transformed counts per 10,000 with the pseudocount of 1 (log2 (CP10k + 1)) using Seurat

(3.1.4).
Dimension reduction analysis of scRNA-seq data based on TE expression profile was performed according to the Seurat flamework (https://satijalab.org/seurat/). Expression level of TEs was normalized by sctransform. Most variably expressed 100 TE subfamilies were selected by the FindVariableFeatures command. After scaling and whitening data, dimension reduction analysis was performed using Uniform Manifold Approximation and Projection (UMAP). The first 10 principle components were used in the analysis.
ATAC-seq data were downloaded from the NCBI Sequence Read Archive (SRA) and mapped to the human reference genome (GRCh38-3.0.0) using BWA Mem (0.7.17) with the default parameters65. Duplicated reads were removed using Picard MarkDuplicates (2.18.6) (https://broadinstitute.github.io/picard/). ATAC-seq peaks were called using MACS2 (2.2.6) with the default parameters. The top 50,000 called peaks were used for downstream analysis. Fold enrichment values for the overlaps between ATAC-seq peaks and a specific subfamily of TEs was calculated using an in-house script (calc_enrichment_randomized.py [https://github.com/TheSatoLab/TE_scRNA-Seq_analysis_Hwang_et_al/).

For manuscripts utilizing custom algorithms or software that are central to the research but not yet described in published literature, software must be made available to editors and reviewers. We strongly encourage code deposition in a community repository (e.g. GitHub). See the Nature Research guidelines for submitting code & software for further information.

Data

Policy information about availability of data

All manuscripts must include a data availability statement. This statement should provide the following information, where applicable:
- Accession codes, unique identifiers, or web links for publicly available datasets
- A list of figures that have associated raw data
- A description of any restrictions on data availability

Accession numbers for the data generated in this study and for the published data used in this study are as follows: the scRNA-seq data generated in this study (GSE153819); the scRNA-seq data of migrating, mitotic and mitotic arrest hFGCs (GSE86146) and of neonatal prospermatogonia (GSE124263); and the RNA-seq data of hPGCLCs and xrOvaries (GSE117101). Human infertility genes can be retrieved from Online Inheritance in Man (OMIM) at https://www.omim.org. 9A13 hiPSC lines are available from the corresponding authors upon request. Tissues, sections or cDNA derived from embryos are not available due to restriction in our study protocol. The remaining data are available within the Article, Supplementary Information, or available from the author upon request.

Field-specific reporting

Please select the one below that is the best fit for your research. If you are not sure, read the appropriate sections before making your selection.

☒ Life sciences ☐ Behavioural & social sciences ☐ Ecological, evolutionary & environmental sciences

For a reference copy of the document with all sections, see nature.com/documents/nr-reporting-summary-flat.pdf

Life sciences study design

All studies must disclose on these points even when the disclosure is negative.

Sample size	No method was used to calculate the sample size a priori. Sample size was chosen based on previous experience and standards in the field. Sample size for d77 and d120 xrTestes was determined based on the data collected in d14 xrTestes. The collected hPGCLC-derived cells in xrTestes bore sufficient sample size for scRNA-seq in this study. For the in vivo samples, three individual testis in 2nd trimester were collected and analyzed independently.
Data exclusions	No data was excluded from this study.
Replication	Induction of MLCs and T1LCs were confirmed by IF and FACS analyses in at least 3 biological replicates. For single cell RNA-seq (scRNA-seq) of xrTestis, pooled samples from at least 6 xrTestes induced at the same timing were used. For scRNA-seq of hPGCLCs, two biological replicates were analyzed. For scRNA-seq and IF of human fetal gonads at 17-18 gestational weeks, 3 biological replicates were used.
Randomization	No particular procedure was applied for randomization since there were no experimental groups in this study.
Blinding	No blinding was applied for sampling for in vivo samples, as testes were required to be preselected for particular fetal stages. Culturing of PGCLCs derived from hiPSCs and generation of xrTestes were performed in a non-blinded manner since there were no experimental groups in this study.

Behavioural & social sciences study design

All studies must disclose on these points even when the disclosure is negative.

Study description	*Briefly describe the study type including whether data are quantitative, qualitative, or mixed-methods (e.g. qualitative cross-sectional, quantitative experimental, mixed-methods case study).*
Research sample	*State the research sample (e.g. Harvard university undergraduates, villagers in rural India) and provide relevant demographic information (e.g. age, sex) and indicate whether the sample is representative. Provide a rationale for the study sample chosen. For studies involving existing datasets, please describe the dataset and source.*

Sampling strategy	*Describe the sampling procedure (e.g. random, snowball, stratified, convenience). Describe the statistical methods that were used to predetermine sample size OR if no sample-size calculation was performed, describe how sample sizes were chosen and provide a rationale for why these sample sizes are sufficient. For qualitative data, please indicate whether data saturation was considered, and what criteria were used to decide that no further sampling was needed.*
Data collection	*Provide details about the data collection procedure, including the instruments or devices used to record the data (e.g. pen and paper, computer, eye tracker, video or audio equipment) whether anyone was present besides the participant(s) and the researcher, and whether the researcher was blind to experimental condition and/or the study hypothesis during data collection.*
Timing	*Indicate the start and stop dates of data collection. If there is a gap between collection periods, state the dates for each sample cohort.*
Data exclusions	*If no data were excluded from the analyses, state so OR if data were excluded, provide the exact number of exclusions and the rationale behind them, indicating whether exclusion criteria were pre-established.*
Non-participation	*State how many participants dropped out/declined participation and the reason(s) given OR provide response rate OR state that no participants dropped out/declined participation.*
Randomization	*If participants were not allocated into experimental groups, state so OR describe how participants were allocated to groups, and if allocation was not random, describe how covariates were controlled.*

Ecological, evolutionary & environmental sciences study design

All studies must disclose on these points even when the disclosure is negative.

Study description	*Briefly describe the study. For quantitative data include treatment factors and interactions, design structure (e.g. factorial, nested, hierarchical), nature and number of experimental units and replicates.*
Research sample	*Describe the research sample (e.g. a group of tagged Passer domesticus, all Stenocereus thurberi within Organ Pipe Cactus National Monument), and provide a rationale for the sample choice. When relevant, describe the organism taxa, source, sex, age range and any manipulations. State what population the sample is meant to represent when applicable. For studies involving existing datasets, describe the data and its source.*
Sampling strategy	*Note the sampling procedure. Describe the statistical methods that were used to predetermine sample size OR if no sample-size calculation was performed, describe how sample sizes were chosen and provide a rationale for why these sample sizes are sufficient.*
Data collection	*Describe the data collection procedure, including who recorded the data and how.*
Timing and spatial scale	*Indicate the start and stop dates of data collection, noting the frequency and periodicity of sampling and providing a rationale for these choices. If there is a gap between collection periods, state the dates for each sample cohort. Specify the spatial scale from which the data are taken*
Data exclusions	*If no data were excluded from the analyses, state so OR if data were excluded, describe the exclusions and the rationale behind them, indicating whether exclusion criteria were pre-established.*
Reproducibility	*Describe the measures taken to verify the reproducibility of experimental findings. For each experiment, note whether any attempts to repeat the experiment failed OR state that all attempts to repeat the experiment were successful.*
Randomization	*Describe how samples/organisms/participants were allocated into groups. If allocation was not random, describe how covariates were controlled. If this is not relevant to your study, explain why.*
Blinding	*Describe the extent of blinding used during data acquisition and analysis. If blinding was not possible, describe why OR explain why blinding was not relevant to your study.*

Did the study involve field work? ☐ Yes ☐ No

Field work, collection and transport

Field conditions	*Describe the study conditions for field work, providing relevant parameters (e.g. temperature, rainfall).*
Location	*State the location of the sampling or experiment, providing relevant parameters (e.g. latitude and longitude, elevation, water depth).*
Access & import/export	*Describe the efforts you have made to access habitats and to collect and import/export your samples in a responsible manner and in compliance with local, national and international laws, noting any permits that were obtained (give the name of the issuing authority, the date of issue, and any identifying information).*
Disturbance	*Describe any disturbance caused by the study and how it was minimized.*

Reporting for specific materials, systems and methods

We require information from authors about some types of materials, experimental systems and methods used in many studies. Here, indicate whether each material, system or method listed is relevant to your study. If you are not sure if a list item applies to your research, read the appropriate section before selecting a response.

Materials & experimental systems

n/a	Involved in the study	
☐	☒	Antibodies
☐	☒	Eukaryotic cell lines
☒	☐	Palaeontology and archaeology
☐	☒	Animals and other organisms
☐	☒	Human research participants
☒	☐	Clinical data
☒	☐	Dual use research of concern

Methods

n/a	Involved in the study	
☒	☐	ChIP-seq
☐	☒	Flow cytometry
☒	☐	MRI-based neuroimaging

Antibodies

Antibodies used

Rabbit anti-OCT3/4 Abcam ab19857
Mouse anti-SOX2 R&D Systems MAB2018
Goat anti-NANOG R&D Systems AF1997
Mouse anti-TFAP2C Santa Cruz Biotechnology sc-12762
Goat anti-SOX17 Neuromics GT15094
Mouse anti-OCT3/4 Santa Cruz Biotechnology sc-5279
Chicken anti-GFP Abcam ab13970
Rabbit anti-Sox9 Millipore AB5535
Mouse anti-HSD3B1 Abcam ab55268
Mouse anti-NR2F2 R&D Systems PP-H7147-00
Mouse anti-ACTA2 Abcam ab7817
Goat anti-CD31 R&D Systems AF3628
Rabbit anti-LAMININ Abcam ab11575
Rabbit anti-DDX4 Abcam ab13840
Goat anti-DDX4 R&D Systems AF2030
Rabbit anti-DAZL Abcam ab34139
Mouse anti-MAGEA3 Millipore MABC1150
Rabbit anti-RFP Abcam ab62341
Rabbit anti-Ki67 Abcam ab15580
Mouse anti-5 methylcytosine (5mC) Active Motif 39649
Rabbit anti-MAGEC2 Abcam ab209667
AlexaFluor 488 conjugated doneky anti-rabbit IgG Life Technologies A21206
AlexaFluor 568 conjugated donkey anti-mouse IgG Life Technologies A10037
AlexaFluor 568 conjugated donkey anti-goat IgG Life Technologies A11057
AlexaFluor 647 conjugated donkey anti-goat IgG Life Technologies A21447
AlexaFluor 647 conjugated donkey anti-rabbit IgG Life Technologies A31573
FITC-conjugated donkey anti-chicken IgY Abcam ab63507
SSEA-1 (CD15) microbeads Miltenyi Biotec 130-094-530

Validation

Rabbit anti-OCT3/4 Abcam ab19857: Recommended for IP, ICC/IF, WB of mouse and human. Validated for detecting Oct4 in human ESCs.
Mouse anti-SOX2 R&D Systems MAB2018: Recommended for WB, ICC/IF, Flow Cyt, WB of human, mouse and rat. Validated for detecting SOX2 in human ESCs.
Goat anti-NANOG R&D Systems AF1997: Recommended for WB, ChIP, ICC of human. Validated for detecting NANOG in human ESCs.
Mouse anti-TFAP2C Santa Cruz Biotechnology sc-12762: Recommended for WB, IP, IHC of mouse, rat and human. Validated for detecting TFAP2C in human lung tumor.
Goat anti-SOX17 Neuromics GT15094: Recommended for ICC, WB, ELISA of mouse and human. Validated for detecting SOX17 in endodermal cells derived from human embryonic stem cells.
Mouse anti-OCT3/4 Santa Cruz Biotechnology sc-5279: Recommended for WB, IP, ICC/IF, IHC, Flow Cyt, ELISA of mouse, rat and human. Validated for detecting Oct4 in human adrenal glands.
Chicken anti-GFP Abcam ab13970: Recommended for IHC, WB, ICC/IF of GFP. Validated for detecting GFP expression of lung adenomas in transgenic mice.
Rabbit anti-Sox9 Millipore AB5535: Recommended for IHC, WB, ChIP, ChIP-seq, ICC, IF of human, mouse, rat and chicken. Validated for detecting SOX9 in human prostate cancer.
Mouse anti-HSD3B1 Abcam ab55268: Recommended for Flow Cyt, WB, IHC of human. Validated for detecting HSD3B1 in human placenta.
Mouse anti-NR2F2 R&D Systems PP-H7147-00: Recommended for WB, ELISA, IP, IHC of human and mouse. Validated for detecting

NR2F2 in mouse embryonic brain.
Mouse anti-ACTA2 Abcam ab7817: Recommended for ICC/IF, IHC, WB, Flow Cyt of mouse, rat, rabbit, human and pig. Validated for detecting ACTA2 in mouse intestine tissue.
Goat anti-CD31 R&D Systems AF3628: Recommended for WB, Flow Cyt, IHC, ICC/IF of mouse and rat. Validated for detecting CD31 in human breast ductal carcinoma.
Rabbit anti-LAMININ Abcam ab11575: Recommended for Dot blot, IHC of mouse and human. Validated for detecting LAMININ in E13.5 mouse embryos.
Rabbit anti-DDX4 Abcam ab13840: Recommended for WB, IHC of mouse, rat, cow, human and pig. Validated for detecting DDX4 in human testis.
Goat anti-DDX4 R&D Systems AF2030: Recommended for WB, IHC of human. Validated for detecting DDX4 in human testis.
Rabbit anti-DAZL Abcam ab34139: Recommended for IHC, ICC/IF, WB of mouse, human and common marmoset. Validated for detecting DAZL in human testis.
Mouse anti-MAGEA3 Millipore MABC1150: Recommended for IHC, ICC/IF, WB of RFP and tdTomato. Validated for detecting MAGEA3 in human testis
Rabbit anti-RFP Abcam ab62341: Recommended for IHC, ICC/IF, WB of human. Validated for detecting RFP expression in pituitary gland of mRFP1 gene transgenic rat.
Rabbit anti-Ki67 Abcam ab15580: Recommended for IHC-P, ICC/IF of human. Validated for detecting Ki67 in human spleen.
Mouse anti-5 methylcytosine (5mC) Active Motif 39649: Recommended for MeDIP, ICC/IF, Flow Cyt, IHC, DB of human and other vertebrate. Validated for detecting 5mC in human midbrain.
Rabbit anti-MAGEC2 Abcam ab209667: Recommended for WB, IHC, ICC/IF, Flow Cyt, IP of human. Validated for detecting MAGEC2 in human gastric cancer tissue.
SSEA-1 (CD15) microbeads Miltenyi Biotec 130-094-530: Recommended for MACS of human and mouse. Validated for binding SSEA-1 in human ES or iPS cells.

Eukaryotic cell lines

Policy information about cell lines

Cell line source(s)	TFAP2C-2A-EGFP (AG) male hiPSCs (585B1 1-7 kind gift from Dr. Mitinori Saitou, Kyoto University).
Authentication	585B1 1-7 hiPSCs were confirmed to have an AG allele by PCR genotyping. Cells was confirmed to have normal karyotype (46XY) in the previous study (Sasaki et al. Cell Stem Cell 2015). 585B1 9A13 (AGVTPC) hiPSCs were derived from 585B1 1-7. 9A13 hiPSCs were confirmed to have AGVTPC alleles by PCR genotyping. Karyotypes of the hiPSC line were first screened by counting the numbers of chromosomes identified by DAPI staining. Cell lines bearing 46 chromosomes were further analyzed by G-banding method performed by Cell Line Genetics (Madison, WI).
Mycoplasma contamination	Confirm that all cell lines tested negative for mycoplasma contamination.
Commonly misidentified lines (See ICLAC register)	No commonly misidentified cell lines were used in this study.

Palaeontology and Archaeology

Specimen provenance	*Provide provenance information for specimens and describe permits that were obtained for the work (including the name of the issuing authority, the date of issue, and any identifying information).*
Specimen deposition	*Indicate where the specimens have been deposited to permit free access by other researchers.*
Dating methods	*If new dates are provided, describe how they were obtained (e.g. collection, storage, sample pretreatment and measurement), where they were obtained (i.e. lab name), the calibration program and the protocol for quality assurance OR state that no new dates are provided.*

☐ Tick this box to confirm that the raw and calibrated dates are available in the paper or in Supplementary Information.

Ethics oversight	*Identify the organization(s) that approved or provided guidance on the study protocol, OR state that no ethical approval or guidance was required and explain why not.*

Note that full information on the approval of the study protocol must also be provided in the manuscript.

Animals and other organisms

Policy information about studies involving animals; ARRIVE guidelines recommended for reporting animal research

Laboratory animals	6-8 weeks old timed pregnant ICR female mice (E12.5) were purchased from Charles River (Wilmington, MD). Mice were housed at temperature and humidity ranges of 22 to 24°C and 40 to 60%, with a 12L:12D cycle.
Wild animals	No wild animals were used in this study.
Field-collected samples	No field-collected samples were used in this study.
Ethics oversight	Animal procedures were conducted in compliance with Institutional Animal Care and Use Committee of the University of Pennsylvania.

Note that full information on the approval of the study protocol must also be provided in the manuscript.

Human research participants

Policy information about studies involving human research participants

Population characteristics	Population characteristics were not used in this study.
Recruitment	Fetal testis samples at 17-18 weeks of gestation were obtained from three donors undergoing elective abortion at the hospital of the university of Pennsylvania. Informed consent was obtained from all the human subjects. Potential self-selection bias might exist, for example, the over- or under-representation of individuals of certain socioeconomic status, which could affect the cellular status of testes. Such variables were not controlled due to the lack of identifiable information of the patients.
Ethics oversight	All experimental procedures were approved by IRB (protocol#832470) and Human Stem Cell Research Advisory Committee at the University of Pennsylvania.

Note that full information on the approval of the study protocol must also be provided in the manuscript.

Clinical data

Policy information about clinical studies

All manuscripts should comply with the ICMJEguidelines for publication of clinical research and a completedCONSORT checklist must be included with all submissions.

Clinical trial registration	*Provide the trial registration number from ClinicalTrials.gov or an equivalent agency.*
Study protocol	*Note where the full trial protocol can be accessed OR if not available, explain why.*
Data collection	*Describe the settings and locales of data collection, noting the time periods of recruitment and data collection.*
Outcomes	*Describe how you pre-defined primary and secondary outcome measures and how you assessed these measures.*

Dual use research of concern

Policy information about dual use research of concern

Hazards

Could the accidental, deliberate or reckless misuse of agents or technologies generated in the work, or the application of information presented in the manuscript, pose a threat to:

No	Yes	
☐	☐	Public health
☐	☐	National security
☐	☐	Crops and/or livestock
☐	☐	Ecosystems
☐	☐	Any other significant area

Experiments of concern

Does the work involve any of these experiments of concern:

No	Yes	
☐	☐	Demonstrate how to render a vaccine ineffective
☐	☐	Confer resistance to therapeutically useful antibiotics or antiviral agents
☐	☐	Enhance the virulence of a pathogen or render a nonpathogen virulent
☐	☐	Increase transmissibility of a pathogen
☐	☐	Alter the host range of a pathogen
☐	☐	Enable evasion of diagnostic/detection modalities
☐	☐	Enable the weaponization of a biological agent or toxin
☐	☐	Any other potentially harmful combination of experiments and agents

ChIP-seq

Data deposition

☐ Confirm that both raw and final processed data have been deposited in a public database such as GEO.

☐ Confirm that you have deposited or provided access to graph files (e.g. BED files) for the called peaks.

Data access links<br>*May remain private before publication.*	*For "Initial submission" or "Revised version" documents, provide reviewer access links. For your "Final submission" document, provide a link to the deposited data.*
Files in database submission	*Provide a list of all files available in the database submission.*
Genome browser session<br>(e.g. UCSC)	*Provide a link to an anonymized genome browser session for "Initial submission" and "Revised version" documents only, to enable peer review. Write "no longer applicable" for "Final submission" documents.*

Methodology

Replicates	*Describe the experimental replicates, specifying number, type and replicate agreement.*
Sequencing depth	*Describe the sequencing depth for each experiment, providing the total number of reads, uniquely mapped reads, length of reads and whether they were paired- or single-end.*
Antibodies	*Describe the antibodies used for the ChIP-seq experiments; as applicable, provide supplier name, catalog number, clone name, and lot number.*
Peak calling parameters	*Specify the command line program and parameters used for read mapping and peak calling, including the ChIP, control and index files used.*
Data quality	*Describe the methods used to ensure data quality in full detail, including how many peaks are at FDR 5% and above 5-fold enrichment.*
Software	*Describe the software used to collect and analyze the ChIP-seq data. For custom code that has been deposited into a community repository, provide accession details.*

Flow Cytometry

Plots

Confirm that:

☒ The axis labels state the marker and fluorochrome used (e.g. CD4-FITC).

☒ The axis scales are clearly visible. Include numbers along axes only for bottom left plot of group (a 'group' is an analysis of identical markers).

☒ All plots are contour plots with outliers or pseudocolor plots.

☒ A numerical value for number of cells or percentage (with statistics) is provided.

Methodology

Sample preparation	The d5 floating aggregates containing hPGCLCs were incubated with 0.1% Trypsin-EDTA (Gibco) in PBS for 15 min at 37 °C with periodical pipetting. After quenching the reaction by addition of FBS and pipetting, cells were strained through a 70 µm nylon cell strainer (Thermo Fisher Scientific). The AG+ cells were sorted by FACSAria Fusion (BD Biosciences) and collected in an Eppendorf tube containing alpha-MEM.<br>For the analysis and the sorting of the xrTestes-derived GCs, xrTestes were treated with 0.1% Trypsin-EDTA in PBS for 15 min at 37 °C with periodical pipetting. After quenching the reaction by addition of FBS and pipetting, cell suspension was washed with 0.1% BSA fraction V in PBS and strained through a 70 µm nylon cell strainer. AG+/-VT+/-PC+/- cells were sorted by FACSAria Fusion and collected in CELLOTION (Amsbio).
Instrument	FACSAria Fusion (BD Biosciences)
Software	FACSDiva software (v.8.0.2) (BD Biosciences)
Cell population abundance	Abundances (percentages) of each population including eGFP, tdTomato and eCFP are indicated for the relevant populations on flow cytometry plots.
Gating strategy	After excluding of dead cells by FSC/SSC gates, doublet discrimination by FSC-A/FSC-H gates, and excluding of auto-fluorescent population by APC, hiPSC or hPGCLC-derived cells expressing eGFP / tdTomato / eCFP fluorescence were gated.

☒ Tick this box to confirm that a figure exemplifying the gating strategy is provided in the Supplementary Information.

Magnetic resonance imaging

Experimental design

Design type	*Indicate task or resting state; event-related or block design.*
Design specifications	*Specify the number of blocks, trials or experimental units per session and/or subject, and specify the length of each trial or block (if trials are blocked) and interval between trials.*
Behavioral performance measures	*State number and/or type of variables recorded (e.g. correct button press, response time) and what statistics were used to establish that the subjects were performing the task as expected (e.g. mean, range, and/or standard deviation across subjects).*

Acquisition

Imaging type(s)	*Specify: functional, structural, diffusion, perfusion.*
Field strength	*Specify in Tesla*
Sequence & imaging parameters	*Specify the pulse sequence type (gradient echo, spin echo, etc.), imaging type (EPI, spiral, etc.), field of view, matrix size, slice thickness, orientation and TE/TR/flip angle.*
Area of acquisition	*State whether a whole brain scan was used OR define the area of acquisition, describing how the region was determined.*

Diffusion MRI ☐ Used ☐ Not used

Preprocessing

Preprocessing software	*Provide detail on software version and revision number and on specific parameters (model/functions, brain extraction, segmentation, smoothing kernel size, etc.).*
Normalization	*If data were normalized/standardized, describe the approach(es): specify linear or non-linear and define image types used for transformation OR indicate that data were not normalized and explain rationale for lack of normalization.*
Normalization template	*Describe the template used for normalization/transformation, specifying subject space or group standardized space (e.g. original Talairach, MNI305, ICBM152) OR indicate that the data were not normalized.*
Noise and artifact removal	*Describe your procedure(s) for artifact and structured noise removal, specifying motion parameters, tissue signals and physiological signals (heart rate, respiration).*
Volume censoring	*Define your software and/or method and criteria for volume censoring, and state the extent of such censoring.*

Statistical modeling & inference

Model type and settings	*Specify type (mass univariate, multivariate, RSA, predictive, etc.) and describe essential details of the model at the first and second levels (e.g. fixed, random or mixed effects; drift or auto-correlation).*
Effect(s) tested	*Define precise effect in terms of the task or stimulus conditions instead of psychological concepts and indicate whether ANOVA or factorial designs were used.*

Specify type of analysis: ☐ Whole brain ☐ ROI-based ☐ Both

Statistic type for inference (See Eklund et al. 2016)	*Specify voxel-wise or cluster-wise and report all relevant parameters for cluster-wise methods.*
Correction	*Describe the type of correction and how it is obtained for multiple comparisons (e.g. FWE, FDR, permutation or Monte Carlo).*

Models & analysis

n/a	Involved in the study
☐	☐ Functional and/or effective connectivity
☐	☐ Graph analysis
☐	☐ Multivariate modeling or predictive analysis

Functional and/or effective connectivity	*Report the measures of dependence used and the model details (e.g. Pearson correlation, partial correlation, mutual information).*
Graph analysis	*Report the dependent variable and connectivity measure, specifying weighted graph or binarized graph, subject- or group-level, and the global and/or node summaries used (e.g. clustering coefficient, efficiency, etc.).*

Multivariate modeling and predictive analysis

Specify independent variables, features extraction and dimension reduction, model, training and evaluation metrics.
